# Supplementary material for: Overexpression of HbGRF4 or HbGRF4-HbGIF1 Chimera Improves the Efficiency of Somatic Embryogenesis in Hevea brasiliensis
Source: Int J Mol Sci. 2024 Mar 2;25(5):2921. doi: 10.3390/ijms25052921 (PMC10932091; doi:10.3390/ijms25052921)
Supplement: Supplementary file 1 [file ijms-25-02921-s001.zip › ijms-2891547-supplementary-Figures.pdf]

Overexpression of *HbGRF4* or *HbGRF4-HbGIF1* Chimera Improves the Efficiency of Somatic Embryogenesis in *Hevea brasiliensis*

Xiaomei Luo [1,2,†], Yi Zhang [1,2,3,4,†], Miaomiao Zhou [1,2], Kaiye Liu [1,2,3,4], Shengmin Zhang [1,2,3,4], De Ye [1,2,3,4], Chaorong Tang [1,2,3,4,*] and Jie Cao [1,2,3,4,*]

[1] School of Breeding and Multiplication (Sanya Institute of Breeding and Multiplication), Hainan University, Sanya 572025, China; luoxiaomei@hainanu.edu.cn (X.L.); 182714@hainanu.edu.cn (Y.Z.); zhoumiaomiao@hainanu.edu.cn (M.Z.); kaiyeliu@hainanu.edu.cn (K.L.); zsm@hainanu.edu.cn (S.Z.); yede@cau.edu.cn (D.Y.)

[2] School of Tropical Agriculture and Forestry, Hainan University, Sanya 572025, China

[3] National Key Laboratory for Biological Breeding of Tropical Crops, Hainan University, Haikou 570228, China

[4] Natural Rubber Cooperative Innovation Center of Hainan Province and Ministry of Education of PRC, Hainan University, Haikou 570228, China

* Correspondence: chaorongtang@hainanu.edu.cn (C.T.); caojie@hainanu.edu.cn (J.C.)

† These authors contributed equally to this work.

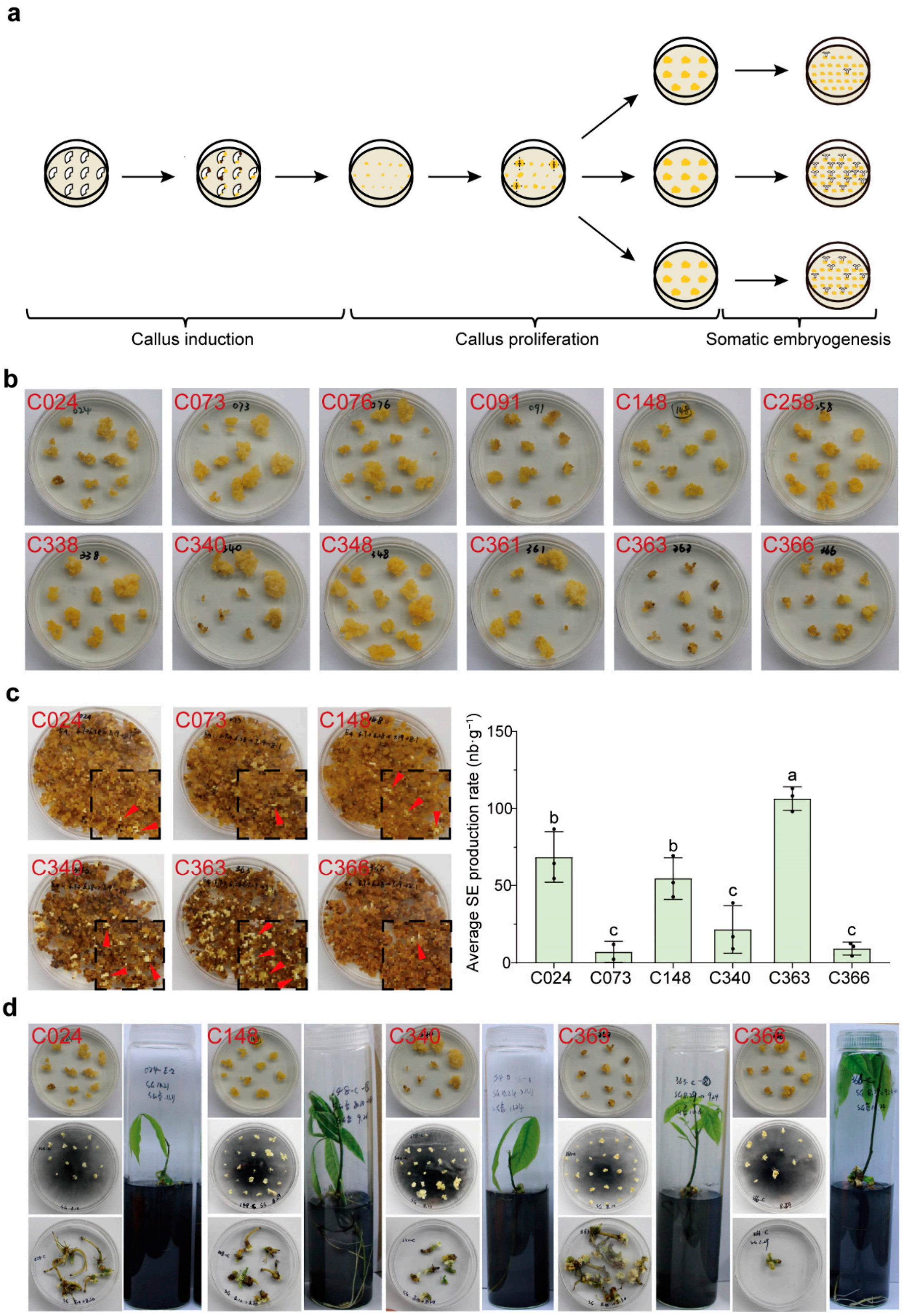

Figure S1. Selection of embryogenic fragile callus lines. **a** The sketch map of callus induction, proliferation and somatic embryogenesis. **b** The phenotypes of fragile callus lines. **c** The SE regeneration rates of fragile callus lines are variable. Arrows indicate somatic embryos. The average SE regeneration rates (nb.g^{-1}) are calculated as the

number of total embryos/gram of calli. Results from individual experiments are indicated by black spots. Letter above each column represents pairwise significant differences. Significant analysis is performed with the ANOVA followed by the Student-Newman-Keuls (SNK) test, $\alpha = 0.05$. **d** Plant regeneration from different fragile callus lines.

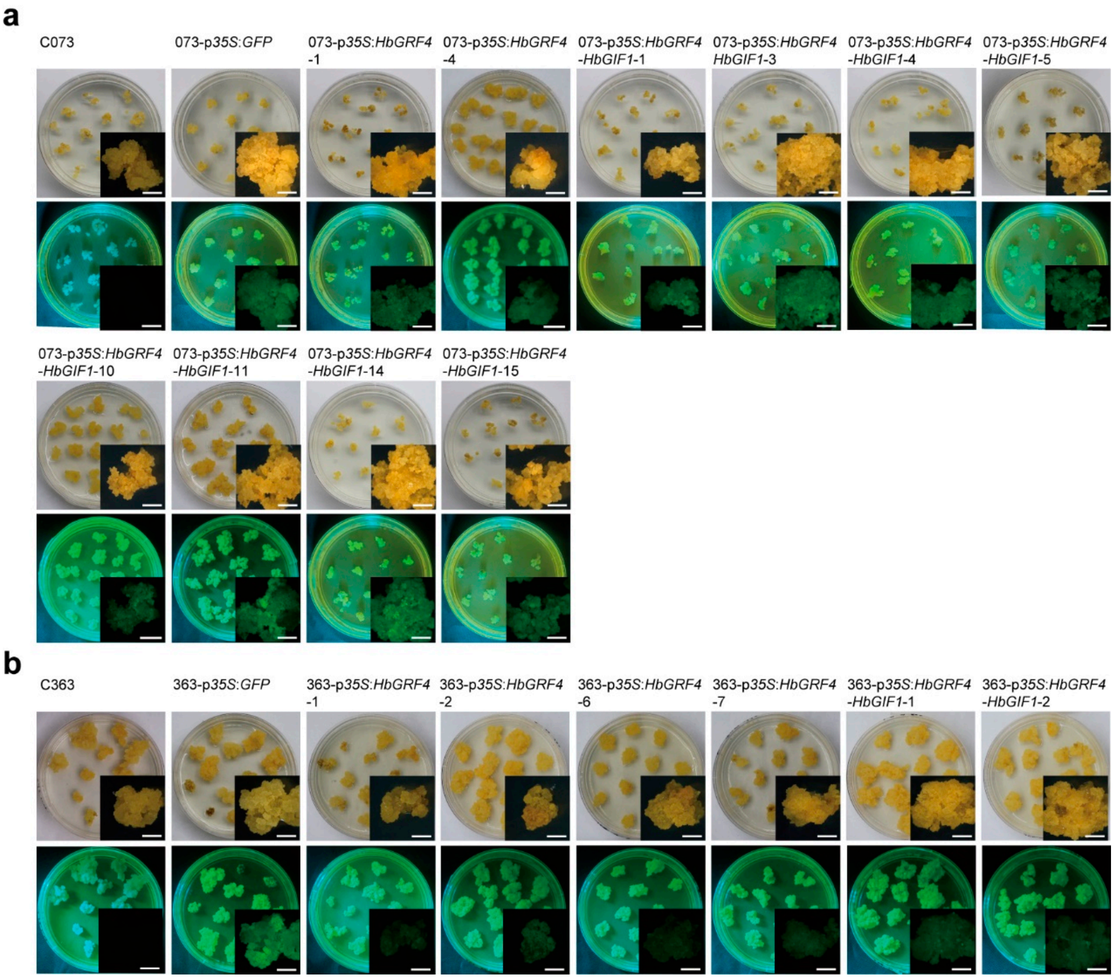

Figure S2. Paromomycin-resistant and homogeneously GFP- fluorescent callus in *HbGRF4*- and *HbGRF4-HbGIF1*-overexpressing lines and controls. **a** Paromomycin-resistant and homogeneously GFP- fluorescent callus in 073-p*35S*:*HbGRF4*-3 and 073-p*35S*:*HbGRF4-HbGIF1*-1 line compare to 073-p*35S*:*GFP*. **b** Paromomycin-resistant and homogeneously GFP- fluorescent callus in 363-p*35S*:*HbGRF4*-3 and 363-p*35S*:*HbGRF4-HbGIF1*-1 line compare to 363-p*35S*:*GFP*. Bar=2 mm.

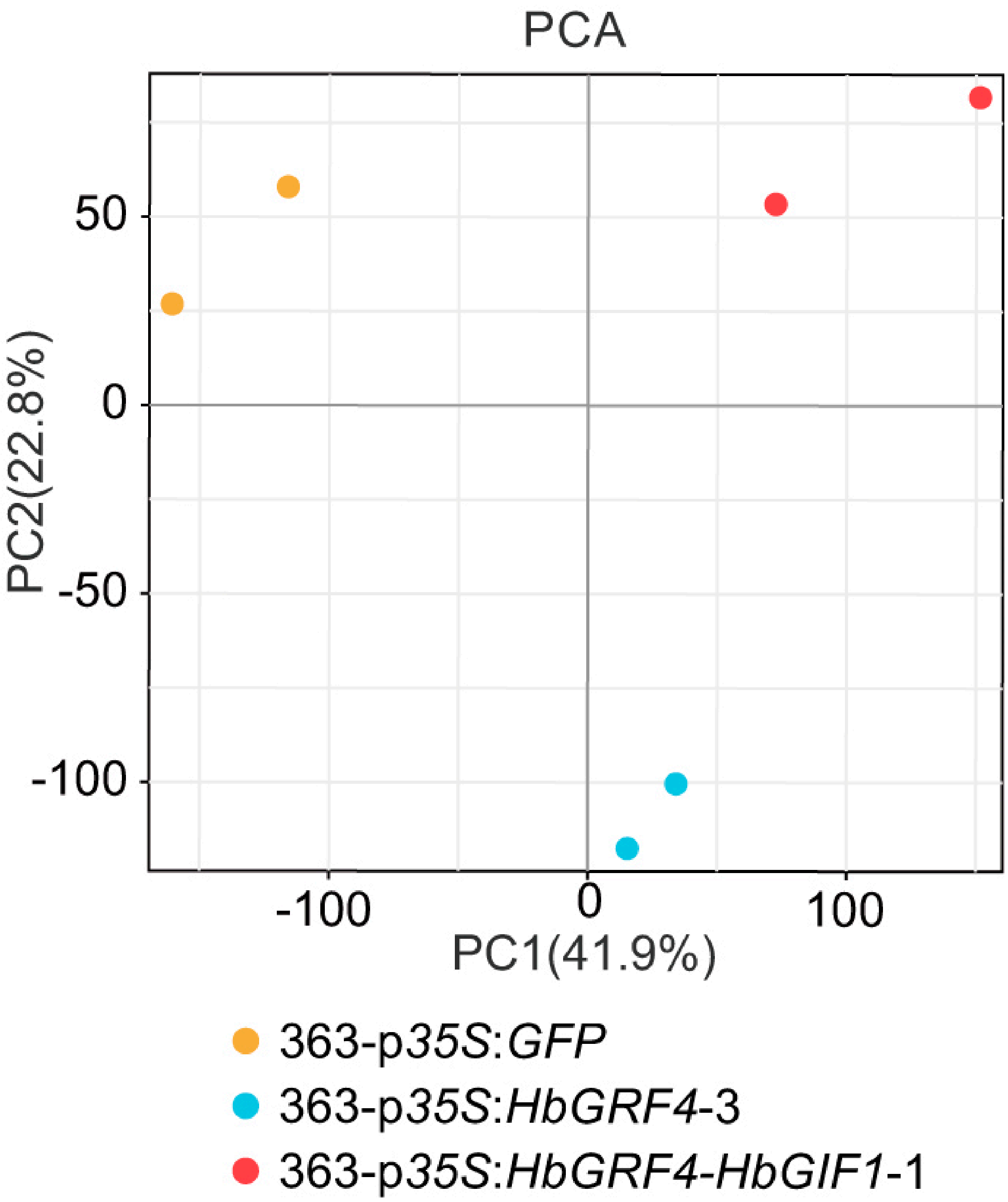

Figure S3. The PCA analysis of DEGs in 363-p*35S*:*GFP*, 363-p*35S*:*HbGRF4*-3 and 363-p*35S*:*HbGRF4-HbGIF1*-1.
